# Social Media Use Among Parents and Women of Childbearing Age in the US

**DOI:** 10.5888/pcd20.220194

**Published:** 2023-02-16

**Authors:** Molly E. Waring, Loneke T. Blackman Carr, Grace E. Heersping

**Affiliations:** 1 Department of Allied Health Sciences, University of Connecticut, Storrs, Connecticut; 2 UConn Center of mHealth & Social Media, University of Connecticut, Storrs, Connecticut; 3 Department of Nutritional Sciences, University of Connecticut, Storrs, Connecticut

## Abstract

Many parents and pregnant women in the US use social media to access health-related information. Estimates of current use of different platforms among these populations are needed. We used data from a 2021 Pew Research Center survey to describe use of commercial social media platforms by US parents and US women aged 18 to 39 years. Most US parents and women of childbearing age use YouTube, Facebook, and Instagram, with most engaging daily. Understanding social media use patterns can help public health professionals, health care systems, and researchers reach selected populations with evidence-based health information and health promotion programs.

**Summary**

**What is already known on this topic?**

The Pew Research Center provided a snapshot of social media use among US adults in 2021. However, the report did not describe social media use patterns among parents or women of childbearing age.

**What is added by this report?**

We used data from a 2021 Pew Research Center survey to describe use of commercial social media platforms by US parents and US women aged 18 to 39 years. Most US parents and women of childbearing age use YouTube, Facebook, and Instagram, with most engaging daily.

**What are the implications for public health practice?**

Understanding social media use patterns can inform efforts to reach target populations with evidence-based health information and health promotion programs.

## Objective

Many parents and pregnant women in the US use social media to access information and support related to parenting and children’s health (1). Misinformation related to maternal and child health is prevalent on social media (2–5) and may be harmful when people use this information to make health-related decisions for themselves and their families. Understanding social media use patterns can help public health professionals, health care systems, and researchers reach selected populations with evidence-based health information and health promotion programs (6,7).

The Pew Research Center provided a snapshot of social media use among US adults in 2021 and documented higher rates of use among younger adults and differences by gender (8). However, the report did not describe social media use patterns at specific stages in the life course, nor among parents or women who may become pregnant, people whose social media use patterns may change (9). A 2014 Pew Research Center survey found that 74% of online parents in the US use Facebook and roughly one-quarter use Instagram, Pinterest, and Twitter, with higher use among women than among men (1). To provide more current estimates of use, we used data from the 2021 survey to describe social media use among US parents and US women of childbearing age.

## Methods

We conducted a secondary analysis of data from the Pew Research Center’s January 2021 Core Trends Survey (8). Telephone surveys were conducted in English or Spanish with adults (aged ≥18) from all 50 US states and Washington, DC, from January 25, 2021, through February 8, 2021 (8). The sample included a combination of landline and cellular telephone random-digit-dialed samples. Participants who provided a mailing address were sent $5. Additional details about the survey methods are available elsewhere (8). This secondary analysis did not require institutional review board approval.

Participants were provided a list of social media platforms and asked whether they ever used any of the individual platforms (yes vs no). Participants who reported using YouTube, Facebook, Instagram, Snapchat, and/or Twitter were asked how often they visit or use the platform (several times a day, about once a day, a few times a week, every few weeks, less often). Participants were asked whether they are the parent or guardian of any children younger than 18 years now living in their household; those who responded yes were considered parents (N = 351). Participants reported whether they described themselves as a man, a woman, or in some other way. Because more than 96% of US births are to women younger than 40 years (10), we considered women aged 18 to 39 years (n = 147) to be of childbearing age. We categorized age and education as in previous research (8). Pew Research Center classified participants as living in urban, suburban, or rural areas based on landline location or respondent-reported zip code for cellular telephone respondents.

First, we described use of each social media platform by parents overall, and by demographic characteristics. Next, we described frequency of platform use among parents. Finally, we described use by women aged 18 to 39 years, overall and by parenting status and age. Analyses were weighted to be representative of US parents or US women aged 18 to 39 years (8). Analyses were conducted using SAS version 9.4 (SAS Institute, Inc).

## Results

The most popular social media platforms among US parents were YouTube (88%; 95% CI, 84%–92%), Facebook (79%; 95% CI, 74%–84%), and Instagram (47%; 95% CI, 42%–53%) (Table 1). Mothers appeared more likely to use Facebook, Instagram, Pinterest, Snapchat, and TikTok while fathers appeared more likely to use Twitter and Reddit. Younger parents appeared more likely to use several platforms, especially Instagram, Snapchat, and TikTok, but appeared less likely to use Pinterest. US parents with at most a high school education and those living in rural areas appeared less likely to use several platforms (Table 1).

**Table 1:** Use of Social Media Platforms Among US Parents, by Demographic Characteristics^a^, Pew Research Center January 2021 Core Trends Survey

Characteristic	YouTube	Facebook	Instagram	Pinterest	WhatsApp	Snapchat	Twitter	TikTok	Reddit
	Weighted % (95% CI)								
**All parents**	88 (84–92)	79 (74–84)	47 (42–53)	37 (31–42)	32 (26–37)	27 (22–32)	27 (22–32)	25 (20–30)	17 (13–21)
**Gender**									
Woman	88 (82–94)	87 (82–93)	52 (44–60)	51 (43–60)	30 (22–38)	32 (24–40)	22 (16–29)	29 (21–37)	13 (7–18)
Man	90 (85–95)	70 (63–78)	43 (35–51)	21 (14–27)	35 (27–43)	21 (14–28)	33 (26–41)	20 (14–27)	22 (16–29)
**Age, y**									
18–29^b^	87 (72–100)	80 (63–97)	70 (50–90)	21 (2–39)	34 (11–56)	69 (49–88)	46 (23–68)	72 (53–91)	15 (2–28)
30–39	92 (86–97)	83 (76–90)	55 (45–65)	35 (26–45)	26 (18–35)	26 (18–35)	27 (19–36)	22 (13–30)	25 (16–33)
40–49	93 (88–98)	77 (69–85)	42 (33–51)	42 (33–51)	33 (24–42)	23 (15–30)	29 (20–37)	22 (14–30)	12 (7–17)
≥50	79 (68–90)	74 (63–86)	35 (23–47)	43 (30–55)	40 (28–53)	16 (7–24)	19 (10–28)	17 (8–27)	12 (4–20)
**Education**									
High school or less	75 (66–85)	71 (61–81)	34 (23–45)	20 (11–30)	29 (18–40)	17 (8–25)	14 (6–22)	24 (14–33)	9 (2–15)
Some college or associate’s degree	95 (91–99)	84 (77–92)	58 (47–68)	42 (32–53)	25 (16–34)	42 (31–53)	30 (21–40)	28 (18–38)	20 (11–28)
Bachelor’s degree or higher	95 (91–98)	82 (76–88)	52 (44–60)	48 (40–56)	40 (33–48)	24 (17–30)	37 (30–45)	23 (17–30)	22 (16–29)
**Rurality of residence**									
Urban	93 (88–99)	79 (71–87)	47 (37–58)	31 (22–40)	37 (27–47)	30 (20–39)	24 (16–32)	30 (21–40)	13 (7–20)
Suburban	89 (83–95)	83 (76–89)	54 (45–62)	40 (31–48)	34 (26–42)	26 (18–34)	30 (22–38)	24 (17–32)	25 (17–33)
Rural	82 (71–92)	75 (63–86)	34 (21–47)	44 (31–57)	15 (5–26)	23 (12–34)	28 (16–40)	18 (7–30)	9 (2–16)

a N = 351 parents. Respondents who did not provide an answer were not included (sex, n = 3; age, n = 9; education, n = 1; urban–rural classification, n = 21; Facebook use, n = 1; Instagram use, n = 1; Pinterest use, n = 1; Reddit use, n = 1). Participants reporting gender identity other than a man or a woman (n = 2) were not included in analyses of gender; these individuals were included in other analyses.

b Only 23 parents in the sample were aged 18 to 29 years; these estimates should be interpreted with caution.

More than half of US parents who use YouTube, Facebook, Instagram, and Snapchat engage on these platforms daily (Figure). Among US parents who use Facebook, 83% (95% CI, 77%–89%) of mothers and 76% (95% CI, 68%–84%) of fathers engage on this platform daily. Among US parents who use Instagram, 50% (95% CI, 38%–62%) of mothers and 54% (95% CI, 41%–67%) of fathers engage daily. 

**Figure:**
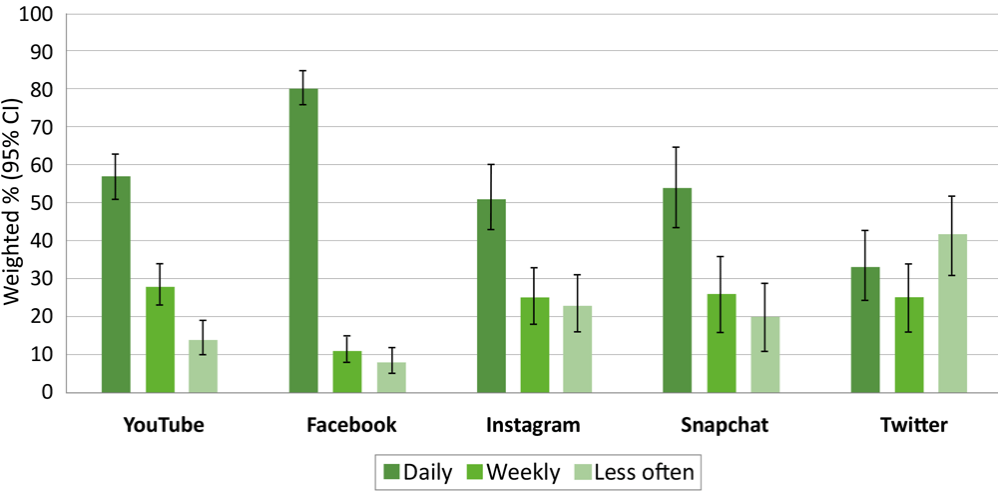
How often US parents visit or use selected social media platforms. Respondents who did not provide an answer for frequency of platform use (YouTube, n = 2; Facebook, n = 1) are not shown. Response options for frequency of use were collapsed as daily (several times a day, about once a day), weekly (a few times a week), or less often (every few weeks, less often). Values are weighted % (95% CI). Source: Pew Research Center’s January 2021 Core Trends Survey (8).

**Table:** 

Social media platform	Weighted % (95% CI)		
	Daily	Weekly	Less often
Twitter	33 (24–43)	25 (16–34)	42 (31–52)
Snapchat	54 (42–65)	26 (16–36)	20 (11–29)
Instagram	51 (43–60)	25 (18–33)	23 (16–31)
Facebook	80 (76–85)	11 (8–15)	8 (5–12)
YouTube	57 (51–63)	28 (23–34)	14 (10–19)

Most US women aged 18 to 39 years used YouTube (92%; 95% CI, 87%–97%), Facebook (84%; 95% CI, 78%–90%), and Instagram (65%; 95% CI, 56%–73%) (Table 2). US mothers aged 18 to 39 years appeared more likely to use Facebook and less likely to use Snapchat, Twitter, TikTok, and Reddit than women aged 18 to 39 years who are not parents. Women aged 18 to 29 years appeared more likely to use Instagram, Snapchat, Twitter, and TikTok (Table 2). Among US women 18 to 39 years who used the respective platform, 60% (95% CI, 51%–68%) used YouTube daily, 75% (95% CI, 67%–83%) used Facebook daily, and 68% (95% CI, 58%–70%) used Instagram daily.

**Table 2:** Use of Social Media Platforms Among US Women Aged 18 to 39 Years^a^, Overall and by Parenting Status and Age, Pew Research Center January 2021 Core Trends Survey

Social media platform	All women aged 18–39 y	Parenting status		Age, y	
		Parent	Not a parent	18–29	30–39
	Weighted % (95% CI)				
YouTube	92 (87–97)	91 (84–99)	93 (87–98)	91 (84–98)	93 (87–99)
Facebook	84 (78–90)	91 (83–99)	79 (70–88)	83 (73–93)	85 (77–93)
Instagram	65 (56–73)	58 (45–71)	69 (59–80)	77 (66–88)	54 (43–65)
Pinterest	47 (39–56)	43 (30–56)	50 (39–61)	54 (42–67)	41 (30–52)
WhatsApp	26 (19–34)	27 (15–39)	26 (16–36)	25 (14–37)	27 (17–37)
Snapchat	52 (44–61)	41 (28–54)	60 (50–71)	78 (67–89)	30 (20–41)
Twitter	31 (24–39)	22 (11–33)	38 (27–49)	45 (32–57)	20 (11–28)
TikTok	41 (33–49)	35 (23–48)	45 (34–56)	58 (45–70)	27 (17–36)
Reddit	23 (16–30)	14 (5–24)	29 (19–39)	26 (15–37)	21 (12–30)

a N = 147 women.

## Discussion

Most US parents use YouTube and Facebook, and nearly half use Instagram. We found social media use to be higher among US parents than US adults generally (8), likely due to greater use among younger adults. For example, while 69% of US adults use Facebook, 79% of US parents and 87% of US mothers use this platform. We saw similar trends among US adults generally in terms of use by gender, age, education, and rurality of residence (8). We found that 80% of US parents who use Facebook engage daily compared with 70% of US adults generally (8). Conversely, 51% of US parents who use Instagram engage daily compared with 59% of US adults (8). We found similar differences by gender among US parents as a report that used data from 2014; use of Instagram and Pinterest has increased over the past 7 years, while use of Facebook and Twitter among US parents was similar (1).

A strength of this study was the use of sampling weights to generate prevalence estimates for US parents and US women aged 18 to 39 years. However, our study also has limitations. Due to the small sample size, we were unable to examine social media use among gender minorities (n = 2) or by race and ethnicity (n = 29 parents identified as non-Hispanic Black, n = 12 as non-Hispanic Asian, and n = 18 as non-Hispanic multiracial or other race). Because only 23 parents in the sample were aged 18 to 29 years, estimates for this group should be interpreted with caution. Because this survey did not ask about the ages of respondents’ children, we cannot describe social media use among parents of younger children (eg, infants, preschoolers) versus older children (eg, teenagers).

Researchers, public health professionals, and health care systems seeking to provide evidence-based health information, combat health misinformation, or deliver behavioral interventions for parents or pregnant women can use information about the social media habits of these target populations when designing their programming and outreach. Platform choice should also be guided by the availability of platforms’ features and functionality (11) and preferences of the target population (12). Most US parents and women of childbearing age use YouTube, Facebook, and Instagram, with most engaging daily. Although only one-quarter of US parents reported using TikTok, use is substantially higher among younger parents and women. Research on health-related topics on TikTok is emerging (5), and TikTok may be a novel platform for reaching younger parents.
